# Multiple sebaceous tumors in a 58‐year‐old man with colorectal cancer

**DOI:** 10.1002/ccr3.5537

**Published:** 2022-03-06

**Authors:** Mohamed Ben Rejeb, Sana Mokni, Maha Ben Rejeb, Nihed Abdelmoula, Baderedine Sriha, Habib Khochteli, Mohamed Denguezli

**Affiliations:** ^1^ 280368 Dermatology Department Farhat Hached University Hospital of Sousse Sousse Tunisia; ^2^ Oral and Maxillofacial Surgery Department Sahloul Univerisity Hospital Sousse Tunisia; ^3^ Anatomopathology Department Hached University Hospital Sousse Tunisia

**Keywords:** colorectal cancer, Muir‐Torre syndrome, sebaceous tumors

## Abstract

The Muir‐Torre Syndrome is a rare genodermatosis, defined by the occurrence of sebaceous neoplasia and internal malignancies and caused by mutations in the mismatch repair gene. We describe the case of 58‐year‐old man who, over the course of several years, had multiple skin lesions and colon cancer. The syndrome was diagnosed using Sanger sequencing, which allowed us to find the causative mutation.

## Graphical abstract

Muir‐Torre syndrome is an autosomal‐dominant genodermatosis characterized by the occurrence of sebaceous neoplasia and visceral malignancy. Colorectal cancer is the commonest visceral malignancy associated with MTS. Metachronous sebaceous gland tumors after colorectal carcinoma along with suspicious family history should prompt genetic analysis for MTS and appropriate counseling of the family members.

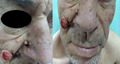

## 1 CASE PRESENTATION

**FIGURE 1:**
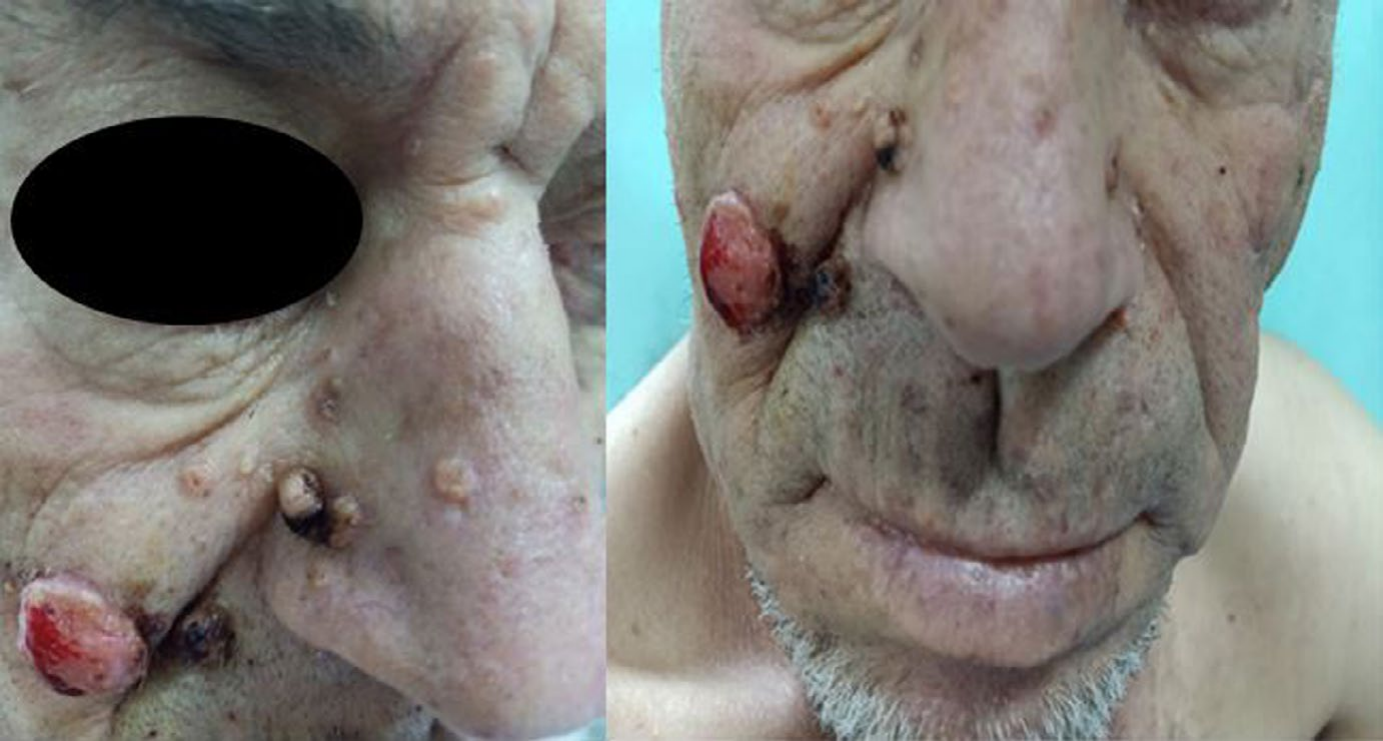
Clinical presentation of sebaceous tumors: numerous yellowish, umbilicated papules, 4–12 mm in diameter over the central and upper face (sebaceous adenomas) with an exophytic erythematous mass on the right cheek (sebaceoma)

We describe a case report of a 58‐year‐old man with a 5‐year history of rectal adenocarcinoma treated with surgery and neoadjuvant radiochemotherapy and a significant family history of 3 cases of colon carcinoma and 1 case of keratoacanthoma. He was referred to our clinic with a 3‐year history of a 2‐cm right cheek exophytic mass associated with multiple eruptive sebaceous adenomas on the face (Figure 1). The patient's tumor and three other sebaceous adenomas were excised. The histopathologic findings confirmed the diagnosis of sebaceous adenoma, and the tumor proved to be sebaceous epithelioma. Germline mutation analysis using Sanger sequencing revealed a heterozygous pathogenic variant *c*.*2361_2362insA* (p.T788NfsX11*)* in exon 14 of *MSH*‐*2* confirming the diagnosis of Muir‐Torre syndrome (MTS).

## 2 DISCUSSION

Muir‐Torre syndrome is an autosomal‐dominant genodermatosis characterized by the association between at least 1 cutaneous sebaceous neoplasm and 1 visceral malignancy, often arising in the gastrointestinal tract.^1^ MTS has been further described as a subtype of Lynch syndrome Type II. Lynch syndrome and MTS are derived from germline mutations in DNA mismatch repair genes, mainly *MLH*‐*1* and *MSH*‐*2*. Sebaceous gland neoplasms are commonly associated with MTS including sebaceous adenomas, sebaceomas, sebaceous carcinomas, and keratoacanthomas.^2^ The early identification of patients presenting with sebaceous skin tumors suggestive of MTS is of the most important in the early diagnosis of internal malignancies.

## CONFLICT OF INTEREST

None.

## AUTHOR CONTRIBUTIONS

Ben Rejeb Mohamed wrote the manuscript and submitted the revised article. Mokni Sana performed the surgical management, collected the images, and revised the manuscript for scientific content. Ben Rejeb Maha conceived the study, performed the surgical management, and revised the manuscript for scientific content. Abdelmoula Nihed searched the literature, designed the study, and preparation of the manuscript. Sriha Baderedine performed the histologic part of manuscript. Khochteli Habib carried out the analysis and corrected the manuscript. Denguezli mohamed supervised and approved the revised manuscript.

## ETHICAL APPROVAL

The ethical statement is approved.

## CONSENT

The consent statement is approved by all authors. Written informed consent was also obtained from the patient and his family to publish this report in accordance with the journal's patient consent policy.

## Data Availability

Data sharing is not applicable—no new data were generated. Data sharing is not applicable to this article as no new data were created or analyzed in this study.
